# Investigation of Concentration and Distribution of Elements in Three Environmental Compartments in the Region of Mitrovica, Kosovo: Soil, Honey and Bee Pollen

**DOI:** 10.3390/ijerph18052269

**Published:** 2021-02-25

**Authors:** Granit Kastrati, Musaj Paçarizi, Flamur Sopaj, Krste Tašev, Trajče Stafilov, Mihone Kerolli Mustafa

**Affiliations:** 1 Faculty of Agribusiness, University of Peja “Haxhi Zeka”, Street, UҪK, 30000 Pejë, Kosovo; granit.kastrati@unhz.eu; 2 Department of Chemistry, Faculty of Mathematics and Natural Sciences, University of Prishtina, Mother Teresa 5, 10000 Prishtina, Kosovo; flamursopaj@gmail.com; 3 State Phytosanitary Laboratory, Bul. Aleksandar Makedonski bb, 1000 Skopje, North Macedonia; tkrste@gmail.com; 4 Institute of Chemistry, Faculty of Natural Sciences and Mathematics, Ss Cyril and Methodius University, Arhimedova 5, 1000 Skopje, North Macedonia; trajcest@pmf.ukim.mk; 5 Department of Environmental Management, International Business College Mitrovica, Bislim Bajgora nn, 40000 Mitrovica, Kosovo; m.kerolli@ibcmitrovica.eu

**Keywords:** Mitrovica, honey, pollen, soil, multivariate analysis, pollution, elements

## Abstract

The abundances of selected elements in different environmental compartments, namely soil, honey, and bee pollen, was determined in this study. For that purpose, sixteen soil and honey samples, and nine pollen samples were taken in the region of Mitrovica, Kosovo. The concentration of elements was measured by ICP-AES and ICP-MS. Pollution level concentrations of Pb, Zn, As, and Cd were observed in soil. The level of soil pollution was estimated by calculating pollution indices. Pb was also observed at high concentrations in honey, as was Cd and Pb in pollen. Pearson’s correlation coefficients revealed mostly weak and moderate correlations of the concentrations of the eight selected elements among the soil, honey, and pollen samples. Several groups of elements with geogenic and anthropogenic origin were identified by hierarchical cluster analysis. The concentrations of selected heavy metals for soil and honey were compared to those in neighboring countries, and those for pollen with samples from Turkey, Serbia, and Jordan.

## 1. Introduction

It is common knowledge that chemical elements are found everywhere in nature, and that they are the building blocks of everything. Every chemical element has an irreplaceable role in nature and the function of its entities. However, under the influence of natural processes or human-driven ones, frequently, certain elements reach sites and concentrations whereby they can have adverse effects on living organisms. It has been shown that metals can induce toxic effects on microorganisms [1,2,3]. Some metals are essential to plants, playing a vital role in their development. However, the toxicity of certain elements, and even that of fundamental nutrients when they surpass certain concentrations, has been observed in various wild and agricultural plants [4,5,6,7,8].

Humans are also intensely exposed to potentially toxic elements because of industrial development. Several researchers have shown various disturbing effects of these elements on human health [9,10,11,12]. They are ingested with food and contaminated water. They can also be inhaled as dust and fine airborne particulate matter, as may be deduced from numerous reports on soil [13,14,15,16,17], water [17,18,19], food [20,21,22], and air pollution [23,24,25].

The subject of this investigation is the concentration of certain elements in soil, honey, and honey bee pollen, with a focus on selected, potentially toxic elements, in the Mitrovica region of Kosovo. The contamination of soils has been reported in many research cases [13,14,15,16,17,26]. Sources of contaminants are of varying nature, e.g., emissions from automobile engines [27,28], the metallurgic industry [14,15,16], the consumption of fossil fuels [29] and other urban processes. Metallic and other elements have been found as contaminants in honey [30,31] and honey bee pollen [32]. Elevated concentrations of potentially toxic elements in soils, as compared to background levels, are probably the most common indicators of pollution [14,15,33]. However, many other components have been considered for monitoring the presence of potentially toxic elements. Among these, honey and honey bee pollen have been put forward as bioindicators. Given that beehives remain in their location for long periods (there are also mobile beehive flocks), they accumulate nectar, water, and pollen from a wide area, i.e., up to 7 km^2^, during foraging. Thus, with some hive flocks, very large areas can be naturally sampled with very good temporal and spatial representation. Although it has been shown that the mineral content of honey is strongly influenced by botanical factors [34], pollution can easily reach it due to anthropogenic activities [34,35]. Pollen is also considered as a bioindicator of pollution containing potentially toxic elements, as it can acquire them through the root systems of plants or through direct atmospheric deposition [32,36].

This study was conducted in the region of Mitrovica, Kosovo, which was expected to be polluted, as the area hosts mines, a lead/zinc smelter, and a battery production plant. In 2000, the lead/zinc smelter was put out of operation, when it was identified as source of significant air pollution [37]; however, previously emitted contamination remains. At present, only mining and ore concentration processes are still performed. The abundances of eighteen elements in soil, multifloral honey and multifloral honeybee pollen were evaluated in the region of Mitrovica; however, this research focuses on eight of them, namely: As, Cd, Co, Cr, Cu, Ni, Pb, and Zn. These elements were selected as they are widely reported as pollutants in the literature, and were expected to be present in the atmosphere of the studied area. An evaluation of the contamination of the area was performed through a statistical analysis of the acquired data. The concentrations of these potentially toxic elements were compared to values found for honey and pollen in articles published from neighboring countries, i.e., Albania, North Macedonia, Montenegro, and Serbia. In the case of pollen, concentrations were compared with those measured in countries such as Turkey, Serbia, and Jordan. Possible correlations regarding selected elements between the soil, honey, and pollen samples were also studied. 

## 2. Materials and Methods

### 2.1. Study Area 

**Figure 1:**
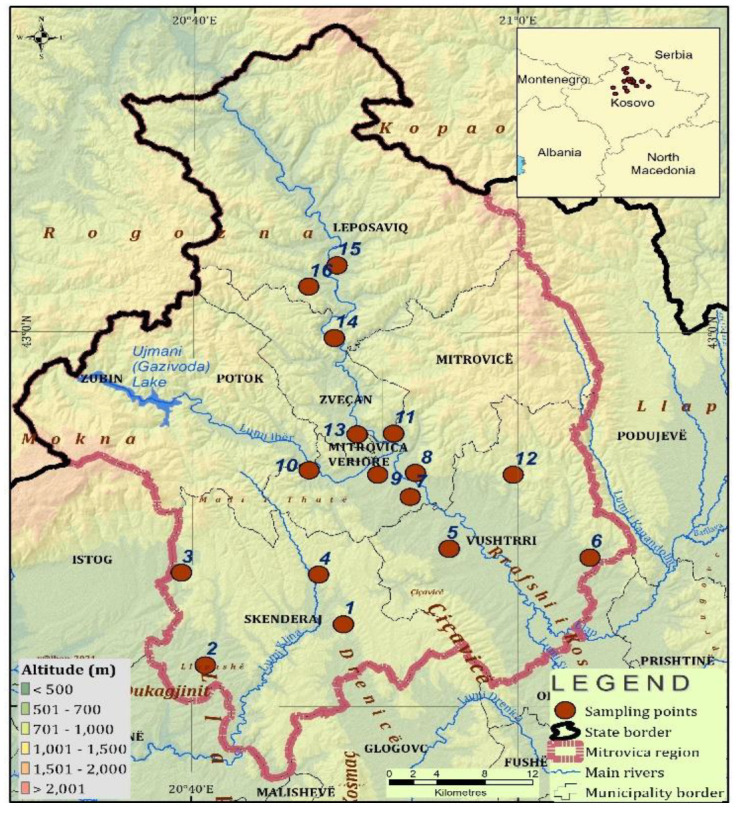
Map of the region of Mitrovica and sample site distribution.

The sampling area was the region of Mitrovica, which is located in the north of Kosovo, and is 2077 km^2^ in size (Figure 1). The sampling locations were numbered as follows: 1 (Polac), 2 (Vajnik), 3 (Padalishtë), 4 (Klinë e epërme), 5 (Bukosh), 6 (Dumnicë), 7 (Frashër), 8 (Shupkofc), 9 (Mitrovicë), 10 (Kushtovë), 11 (Zveçan), 12 (Karaq), 13 (Zveçan), 14 (Kulla), 15 (Krushevle) and 16 (Leposaviç). The samples of soil and honey were collected from 16 locations spread as homogeneously as possible (Figure 1), and honey bee pollen samples were collected in 9 locations (3, 4, 5, 6, 7, 8, 9, 10 and 11). 

### 2.2. Soil, Honey, Pollen Sampling 

At each sampling point representing nonagricultural land, about 1 kg of 3 to 5 soil subsamples in a 50 × 50 m area were taken. The soil samples were put into plastic bags, brought in the laboratory for cleaning and homogenization, and then dried in an oven at 45 °C for three days until constant dry mass was achieved. Once the soil was dried, it was ground; then, subsamples were mixed to obtain a more representative sample, and the obtained mixture was sieved through a 125 μm sieve for better digestion. The sieved soil was put in plastic containers and stored until further treatment. Honey samples were collected directly from beehives using a wooden spoon, and were stored in plastic containers. To collect pollen samples, a plastic grid equipped with a recipient was put at the entrance of each hive, so that the pollen could be combed off the bees into the recipient. Pollen was also stored in plastic containers.

### 2.3. Chemical Analysis

An amount of 0.25 g of each soil sample was put into a Teflon beaker, into which 5 mL 69% HNO_3_ were added. The mixture was then heated until evaporation was complete. After cooling, 5 mL HF and 1.5 mL HClO_4_ were added. Finally, after evaporation and cooling, 1.5 mL HCl and 3 mL distilled water was added into the sample. The solutions were then diluted to 25 mL in a volumetric plastic flask and sent for analysis. 

Honey and pollen samples were digested in a microwave system; 0.5 g of honey or pollen was put in the Teflon tube, into which 7 mL HNO_3_ (69% V/V, trace pure, Merck, Darmstadt, Germany) and 2 mL H_2_O_2_ p.a. (30% V/V, Merck, Darmstadt, Germany) were added, and a microwave digestion system (Analytic Jena TOPwave, Jena, Germany) was applied with the following program: 5 min up to 170 °C, hold time of 10 min at 170 °C, 1 min up to 200 °C, hold time of 15 min at 200 °C, 1 min down to 50 °C, and hold time of 23 min at 50 °C. The solutions obtained were then diluted to 25 mL in a volumetric flask and sent for analysis. 

By the application of inductively coupled plasma–atomic emission spectrometry (ICP-AES, Varian, model 715ES, Palo Alto, CA, USA), the concentrations of the following 13 elements were determined: Al, Ba, Ca, Cr, Cu, Fe, K, Mg, Mn, Na, P, Sr and Zn. Inductively coupled plasma–mass spectrometry (ICP-MS, Plasma Quant ICP-MS, Analytic Jena, Jena, Germany) was applied for the analysis of As, Cd, Co, Ni and Pb. Standard solutions of the analyzed elements were prepared by dilution of 1000 mg/L solutions (11355-ICP multi Element Standard, Merck, Darmstadt, Germany). 

Both certified reference materials (NIST-SRM 2711a, Montana II Soil, National Institute of Standards & Technologies, Gaithersburg, MD, USA) and spiked intralaboratory samples were analyzed at a combined frequency of 20% of the samples. The recovery for all of the analyzed elements ranges from 76.8% for Tl to 119% for Sb (for ICP-MS measurements) and from 87.5% for Na to 112% for P (for ICP-AES measurements).

### 2.4. Methods for Estimating Pollution Indicators

In order to assess the level of pollution, five indices were calculated: contamination factor (*CF*) [16], degree of contamination (*Cd*) [38], modified degree of contamination (*mCd*) [39], pollution load index (*PLI*) [39,40], and geo accumulation index (*Igeo*) [41]. 

The formula used to calculate the contamination factor (*CF*) of each metal in the soil samples was:

(1) $$CF=C_{sample}^{i}/C_{reference}^{i}$$

where *CF* is the contamination factor for heavy metal; C^*i*^_*sample*_ is the measured value of the heavy metal at 0–5 cm depth of soil; and *C*^*i*^_*reference*_ are the parameters for calculation concerning the background values, i.e., the European topsoil averages of potentially toxic elements [42]. According to Hakanson [43], *CF* values may be assigned as follows: *CF* < 1—low contamination factor, 1 ≤ *CF* < 3—moderate contamination factor, 3 ≤ *CF* < 6—considerable contamination factor, and *CF* ≥ 6—very high contamination factor.

The degree of contamination (*Cd*) is defined as the sum of all contamination factors of the investigated elements:

(2) $$Cd=\sum_{n=1}^{n=8}CF_{i}$$

The following classification proposed by Håkanson [43] was adopted to describe the extent of contamination for the analyzed elements: *Cd* < 6: low contamination degree, 6 ≤ *Cd* < 12: moderate contamination degree; 12 ≤ *Cd* < 24: considerable contamination degree; and *Cd* ≥ 24: very high contamination degree.

To provide an overall average value for a range of pollutants, the Modified Degree of Contamination (*mCd*) was calculated using the generalized formula:

(3) $$mC_{d}=\;\frac{\sum_{i=1}^{n=8}CF_{i}}{n}$$

where *n* is the number of analyzed element and *CFi* is the contamination factor. Abrahim [39] proposed the following modified degree of contamination (*mCd*): *mCd* < 1.5—nil to very low degree of contamination, 1.5 ≤ *mCd* < 2—low degree of contamination, 2 ≤ *mCd* < 4—moderate degree of contamination, 4 ≤ *mCd* < 8—high degree of contamination, 8 ≤ *mCd* < 16—very high degree of contamination, 16 ≤ *mCd* < 32—extremely high degree of contamination, and *mCd* ≥ 32—ultra-high degree of contamination.

The *PLI* provides information about the abundance of the analyzed element in the environment. The *PLI* of a single site is the *n* root of *n* number, multiplied with the contamination factor (*CF*) values:

(4) $$PLI=\;{\left(CF1\;x\;CF2\;x\;CF3\;x\ldots CFn\right)}^{\frac{1}{n}}$$

where *CF* is the contamination factor and *n* is the number of metals. When *PLI* is greater than 1, it means that contamination exists, while if *PLI* is less than 1, there is no such contamination.

To assess the contamination impact of potentially toxic elements in the soil, a common approach, the geoaccumulation index (*Igeo)*, was used to calculate the concentrations of elements relative to background or baseline concentrations. The method assesses the degree of metal contamination in terms of seven classes, based on the increasing numerical values of the index. This index is calculated as follows:

(5) $$I_{geo}=\;\frac{log2\;x\;C_{n}}{1.5\;B_{n}}$$

where *Cn* is the concentration of the element in the enriched samples, and *Bn* is the average of European topsoil [42]. Müller proposed the following descriptive classes for increasing Igeo values [41]: *Igeo* > 5—extremely contaminated, *Igeo* from 4 to 5—strongly to extremely contaminated, *Igeo* from 3 to 4—strongly contaminated, *Igeo* from 2 to 3—moderately to strongly contaminated, *Igeo* from 1 to 2—moderately contaminated, *Igeo* from 0 to 1—uncontaminated to moderately contaminated, and Igeo ≤ 0 uncontaminated.

All of the pollution indices in this work were calculated only for the eight selected potentially toxic elements: As, Cd, Co, Cr, Cu, Ni, Pb, and Zn. 

### 2.5. Statistical Analysis

Microsoft Excel and SPSS 12 software (StatSoft. Inc, TULSA, OK, USA) were used to perform statistical analyses. Cluster analysis was also used to investigate the similarities between element patterns from soil, honey, and pollen samples. Evaluations of similarity between groups of cluster analyses were based on the Ward’s method [19].

## 3. Results and Discussions 

### 3.1. Soil

Eighteen elements were evaluated in 16 soil samples collected throughout the region of Mitrovica at depths 0–5 cm and 20–30 cm. To show the concentration of these elements, basic statistical parameters such as mean, median, minimum and maximum concentration are presented in Table 1 and Table 2. The maximum concentrations of eight potentially toxic elements at 0–5 cm depth in mg/kg were as follows: Pb (3610) > Ni (1263) > Zn (1116) > Cr (1099) > As (272) > Cu (88.8) > Co (71.6) > Cd (8.80). The median of concentrations over the whole sampling area for each element followed the order: Pb (166) > Zn (153) > Cr (102) > Ni (94.7) > Cu (35.7) > Co (14.0) > As (8.16) > Cd (0.41). A particularly high concentration of Pb was found in sample 11 (Mitrovicë–Zveçan), which is in accordance with the previous work [14]. Concentrations of over 500 mg/kg of Pb were found at sampling sites 7, 8, and 14, whereas in 10 samples, the concentrations of Pb were over 100 mg/kg. Arsenic concentration was relatively homogenously distributed, except in sample 8, which showed a remarkably high value, i.e., 272 mg/kg. Spikes in concentrations were also observed for Ni and Cr in sample 14, and Zn in sample 11. The high concentrations of Ni and Cr in sample 14 may have been due to vehicle exhaust and wear of vehicle parts from a regional road which almost surrounds it, and also from lead/zinc smelting facilities. However, the geochemical composition of the soil may be the most important reason for the high Ni and Cr concentrations in this location, because high concentrations of these two metals were measured at depths of 20–30 cm. Concentrations over 50 mg/kg were measured for Cu in samples 2, 3, 8, and 11, whereas Co exceeded 50 mg/kg only at sampling point 14; in all other samples, its concentration was 40 mg/kg or less. The highest concentration for Cd was observed at sampling point 8 and 3, with concentrations of 8.8 and 6.6 mg/kg respectively; all other values were about 1 mg/kg or lower. Although the concentration of Cd was much lower than those of other metals, it stood out in comparison to its concentration in other locations, except for that at sampling point 8, which was the highest. This increased concentration may have been a result of the dust transported by wind from a gravel excavation site which is located nearby, in the north.

The concentrations of the selected elements agree in general with those reported in a study by Šajn et al. [14]; however, some discrepancies may have arisen from the fact that the area covered in the present work was larger, and not limited to the area around the lead/zinc smelting site, where higher pollution would be expected. However, just as Šajn et al. [14] indicated, most of the high concentrations of selected potentially toxic elements were found around the lead/zinc smelting site, which is located just beside the city of Mitrovica. It is worth noting that the maximum lead concentration in this region significantly exceeds the maximum concentration allowed for some industrial sites in neighboring countries, namely Albania, Serbia, and North Macedonia [44,45,46]; see Table 1. Arsenic was also found in higher concentrations than in soils surrounding industrial facilities in Vojvodina, Serbia [45], and North Macedonia [46]. However this comparison is relatively accurate, because the dissolution method of soil samples in the aforementioned countries was not the same (aqua regia, and sequential extraction) as in this work (total dissolution). 

**Table 1:** Basic statistics of element concentrations in surface soil, i.e., 0–5 cm (in mg/kg), in Balkan countries.

	Kosovo 2019 (Present Work); *n* = 16				North Macedonia, 2010 [46]; *n* = 344			Albania, 1998 [44]; *n* = 8	Serbia, 2010 [45]; *n* = 45		
	Mean	Median	Range	STD	Mean	Median	Range	Range	Mean	Median	Range
Al	22,608	19,331	8212–46,207	10,427	65,000	6600	900–11,000	-	20,399	20,417	10,444–31,386
As	25.4	8.16	2.01–272	66.2	15	10	1–720	-	6.55	6.52	1.09–21.4
Ba	251	247	157–449	87.0	500	430	6.0–2900	-	-	-	-
Ca	26,570	11.347	2344–146,582	37,474	28,000	13,000	500–350,000	2100–28,000	28,078	4734	24,062–68,422
Cd	1.37	0.41	0.10–8.8	2.53	0.81	0.30	0.10–110	2.0–14	0.36	0.33	0.21–1.27
Co	17.1	14	4.23–71.6	16.0	18	17	0.50–150	130–476	10.8	9.97	5.31–24.1
Cr	175	102	45.7–1099	252	130	88	5.0–2700	91–3865	49.3	36.8	21.1–247
Cu	41	35.7	16.5–88.8	18.7	32	28	1.6–270	6.0–1107	28.0	22.8	13.6–94.4
Fe	24,936	23,287	14,693–45,381	8466	36,000	35,000	300–120,000	-	20,888	21,144	11,515–34,855
K	11,653	11,616	7619–15,812	2614	19,000	19,000	200–53,000	-	1531	1614	391–3013
Mg	7205	5451	2603–20,450	4850	13,000	9400	1200–130,000	10,400–25,300	18,201	13,805	6061–85,900
Mn	742	553	408–1983	407	1000	900	17–10,000	-	630	625	455–899
Na	1868	1998	472–3415	944	13000	12000	130–60,000	-	-	-	-
Ni	199	94.7	29.9–1263	296	78	46	2.1–2500	54–3579	51.6	39.9	23.5–230
P	752	627	524–1673	316	700	620	110–25,900	-	-	-	-
Pb	487	167	15.5–3610	897	95	32	1.2–10,000	80–172	21.6	18.6	6.42–67.7
Sr	87.9	60.9	31.4–2508	62.2	190	140	21–1400	-	-	-	-
Zn	224	153	69.6–1116	254	140	35	0.8–210	49–2495	65.9	51.3	33.4–192

On 28 August 2018, guidelines were approved by the government of Kosovo, i.e., decision No.13/62 [47]. According to these regulations, polluted soil is divided into three categories regarding metal pollution; A—clean, B—acceptable contamination, but further investigation is required, and C—high contamination and needs to be cleaned. The median concentrations of Pb, Zn, Ni, As, Cr measured in this investigation fell into category C; those of Cd and Ni were between A and B, and that of Cu corresponded with category A.

The highest concentrations of Pb and Zn, as well as other metals, were found around the lead/zinc smelting site and the tailing waste dump. This was expected, since ore processing emits fine particles which contain these elements. At sampling points 7, 8, 11, and 14, the concentration of lead corresponded to category C, whereas at sampling points 13, 15, and 16, it fell between category A and B. The rest of the sampling points corresponded to category A. 

Zinc concentrations fell within category B at sample 8, and category C for sample 11. Higher concentrations of Cu were measured at sample points 2 and 3, and for Cd at sample point 3; those locations were the most distant from mineral processing facilities of lead/zinc in Zveçan and Mitrovica, and as such, they corresponded to the category A. Arsenic corresponded to category C only at sampling points 8 and 11, and to category A in the remaining locations. In the case of Cr, all samples corresponded to category A, except 14, which was within category C. Most Co concentrations were within category A, except for those from sampling sites 14 and 15, which were between A and B.

**Table 2:** Basic statistics of element concentrations in the subsurface soil, i.e., 20–30 cm (in mg/kg).

	Al	As	Ba	Ca	Cd	Co	Cr	Cu	Fe	K	Mg	Mn	Na	Ni	P	Pb	Sr	Zn
Mean	22,889	9	286	30,100	1	19	197	35	25,890	12,059	6896	686	1889	219	617	252	100	190
Median	22,023	7	232	12,399	0.14	13	96	34	24,594	13,026	5315	553	1979	91	527	135	73	130
Min	9962	1	114	1288	0.1	3	50	15	15,354	6513	1763	384	227	22	309	5	27	47
Max	41,202	20	505	152,162	9	97	1429	57	55,976	16,572	24,626	1350	3783	1648	2040	1398	251	497
STD	10,329	5.18	120	41,431	2.29	23.2	344	11	9894	3007	5497	292	1133	403	435	362	71	143

In Table 2, the basic statistical parameters for elements in subsoil samples with a depth of 20–30 cm are shown. The maximum values of selected potentially toxic elements (in mg/kg) in increasing order were: Ni (1648) > Cr (1429) > Pb (1398) > Zn (497) > Cu (57) > As (20) > Co (13) > Cd (9). The median values are Pb (135) > Zn (130) > Cr (96) > Ni (91) > Cu (34) > Co (19) > As (7) > Cd (0.14). The concentration of Pb in soil at a depth of 20–30 cm was lower than at a depth of 0–5 cm. Its highest value, 1398 mg/kg, was measured at location 7, whereas at locations 11, 13, and 14, it was 631, 291, and 447 mg/kg, respectively. The rest of the values were under 200 mg/kg. Unlike at 0–5 cm depth, at 20–30 cm, the As concentration was far lower: in sample 8, its concentration was 6.43 mg/kg; in samples 7, 10, 11, and 16, it ranged between 10 and 20 mg/kg, whereas the rest of the samples had a concentration below 10 mg/kg. Ni and Cr were found at high concentrations at location 14 (at 0–5 cm depth). Zn was found in concentrations between 200–500 mg/kg at five locations in increasing order 7 > 12 > 8 > 11 > 14. Cu was more homogeneously distributed, with most of its concentrations being between 20–40 mg/kg. Its highest concentration was detected at sampling points 7 (57.2 mg/kg) and 2 (54.2 mg/kg). The highest concentration of Co was observed at sample location 14 (97.0 mg/kg); the other values were 35 mg/kg or less. For Cd, only at point 7 was it close to 9 mg/kg; elsewhere, concentrations were 3 mg/kg or below.

The *CF* values for each sampling point for the selected potentially toxic elements are given in Table 3. The *CF* values varied greatly among the samples, from those indicating low contamination, moderate contamination, and highly contaminated. The highest *CF* was found for Pb in sample 11, with a value of 157. Extremely high *CF* were also found for Ni in sample 14 (70.2), Cd in sample 8 (58.7), and As in sample 8 (38.9). 

The contamination degree values are given in Table 4. All samples fell within the *Cd* interval values for considerable or very high contamination degree. The most extreme *Cd* values were observed for samples 11 (206), 8 (175), and 14 (142). As can be seen in Table 4, only sample 6 showed a low modified degree of contamination (*mCd*); then, there were samples with moderate, high, and very high degrees of contamination. Samples 8, 11, and 14 were extremely contaminated, with *mCd*, 21.9, 25.7, and 17.8 respectively. Table 4 also shows that there was no sample with a *PLI* less than 1, which indicated that the whole area of the region of Mitrovica is polluted with potentially toxic elements. The most polluted site was revealed to be location 8, with *PLI* 10.0, followed by 14, with *PLI* 8.26, and 11, with *PLI* 7.83.

According to the *Igeo* classification (Table 5), only samples 11 and 8 appeared to be extremely contaminated with Pb. Samples 14 and 8 were extremely contaminated with Ni and Cd, respectively (Table 5). Sample 8 was strongly contaminated with As. Co, Cd, and As were the least abundant contaminants, followed by Cr and Zn, whereas Ni and Pb had the highest Igeo values.

**Figure 2:**
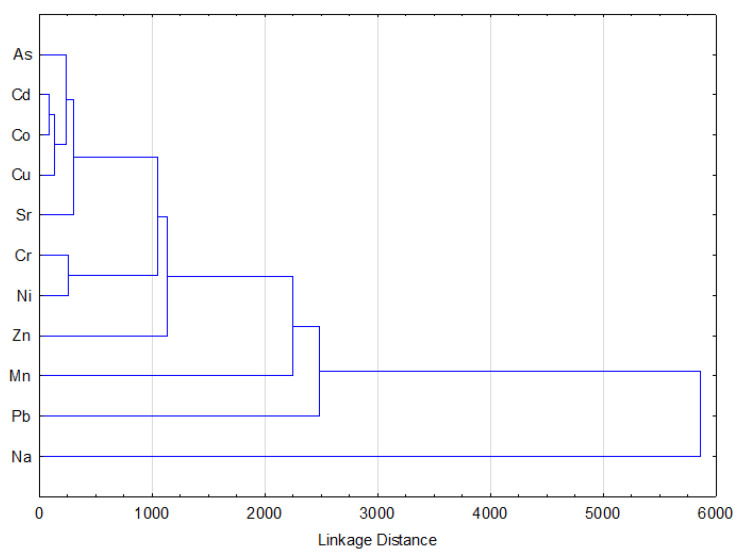
Hierarchical cluster dendrogram for elements in soil.

A hierarchical cluster analysis is presented in Figure 2. The dendrogram constructed by Ward’s method revealed six clusters. The first was associated with Ca, the second with Al and Fe, the third with K and Mg, the forth with Na, the fifth with Zn, Mn, Pb, and the sixth with As, Cd, Co, Cu, Sr, Cr and Ni. Clusters of Ca, Al and Fe, and K and Mg, are not presented in Figure 2, as the aforementioned very high values obscured the data. Clusters 1, 2, 3, 5 and 6 were mostly related to the geochemical composition of the soil [14]. Cluster 5 appeared to be due to the anthropogenic activities, i.e., mainly from the lead smelting sites in Zveçan and Mitrovica (samples 9, 11 and 13). Although elements in cluster 6 may have been of geogenic origin, the concentrations of these elements and the pollution indices indicated that some sites were also polluted with As, Cd, Co, Cr, Ni, and Cu. The increased concentrations of these elements also originated from mineral processing at the aforementioned lead and zinc smelting sites [14,48].

### 3.2. Honey

Multifloral honey samples were collected at the same locations as the soil samples, and after analysis, the maximum concentrations in mg/kg for selected elements were as follows (Table 6): Cu (2.98) > Pb (2.10) > Zn (1.90) > Cr (0.84) > Ni (0.22) > As (0.12) > Cd (0.04) > Co (0.03), and median values: Cu (1.33) > Zn (0.73) > Pb (0.42) > Ni (0.13) > Cr (0.1) > As (0.04) > Cd (0.02) > Co (0.01). Samples from seven sampling locations showed Cu concentrations between 1.52 –2.98 mg/kg; at the other sites, these values were 1.24 mg/kg or lower. A spike in the lead concentration was observed at site 11; five other locations fell within the range of 0.55–1.14 mg/kg, and the concentrations of Cu in the rest of the samples were under 0.5 mg/kg. Samples 6, 7, 8, 10, and 16, had concentrations of Zn between 1.04–1.9 mg/kg, while in the rest of them, it was below 1 mg/kg. Cr spikes were identified in samples 4, 6, 9, 11, with concentrations between 0.37–0.84 mg/kg; at point 3, it was 0.2 mg/kg, and in all other samples, it was 0.1 mg/kg. The highest concentration of As was measured in the sample from location 4, whereas the rest of the concentrations ranged between 0.02–0.06 mg/kg. Most of the Cd concentrations were between 0.01–0.044 mg/kg, while most of the Co concentrations were around 0.01 mg/kg.

The most abundant of the eight selected potentially toxic elements in honey was Cu, with a median of 1.33 mg/kg and a maximum concentration of 2.98 mg/kg. A comparison among three Balkan countries is given below (Table 6). The median for Cu was higher than that measured in honey from the whole territory of North Macedonia, which was 0.69 mg/kg, while the maximum concentration, i.e., 2.98 mg/kg, was lower compared to that measured in North Macedonia, i.e., 5.9 mg/kg [49]. However, the mean concentration of Cu (1.57 mg/kg) was higher than those found in Montenegro [50] and Serbia [51]. Lead mean concentration (0.54 mg/kg) was higher than that found in Serbia (0.0064 mg/kg) [51] and in honey from some locations in Montenegro [50] (with a maximum mean of 0.21 mg/kg). The median value for zinc (0.73 mg/kg) was lower than that of North Macedonia (2.3 mg/kg) [49], whereas the mean concentration was lower than in honey samples from the countries mentioned. The Cr mean value was higher than in Serbia (0.0053 mg/kg) and lower than in some areas in Montenegro. The Ni mean (0.14 mg/kg) was close to that in Serbia (0.129 mg/kg). The As mean concentration in honey from the Mitrovica region was 0.04 mg/kg, i.e., higher than in Serbia. Cd was present with a median of 0.02 mg/kg, which was higher than that in North Macedonia (0.0031 mg/kg) 

**Table 6:** Basic statistics of elements concentration in honey (in mg/kg) in Balkan countries.

	Kosovo, 2019 (Present Work) *n* = 16				North Macedonia, 2007 [49] *n* = 123			Montenegro, 2020 [50] *n* = 24		Serbia, 2010 [51] *n* = 32	
	Mean	Median	Range	STD	Mean	Median	Range	Mean	Range	Mean	Range
Al	2.59	1.99	0.49–9.10	2.09	-	-	-	-	-	-	-
As	0.04	0.04	0.02–0.12	0.02	-	-	-	-	-	0.00168	0.001–0.0054
Ba	0.09	0.07	0.01–0.20	0.06	-	-		0.29	ND−1.44	-	-
Ca	19.06	15.66	8.84–59.07	12.04	51	41	4.1–170	93.25	48.88–152	107.8	17.47–173.4
Cd	0.02	0.02	0.01–0.04	0.01	0.010	0.0031	0.001–0.27	0.02	ND-0.08	0.00265	0.001–0.0064
Co	0.01	0.01	ND–0.03	0.007	-	-	-	-	-	0.0155	0.004–0.078
Cr	0.23	0.10	0.10–0.84	0.24	-	-	-	0.44	0.10–1.13	0.00528	0.002–0.0207
Cu	1.57	1.33	0.52–2.98	0.80	1.4	0.69	0.023–5.9	0.64	0.31–0.98	0.1939	0.06535–0.407
Fe	5.87	4.41	1.93–14.9	3.88	1.9	1.5	0.028–7.0	10.14	3.95–15.93	1.98	0.57–7.02
K	858	775	121–2398	594	1205	1021	169–3323	1617.92	713–2589.33	943.9	334.1–2263
Mg	15.6	14.4	3.22–33.5	8.92	30	15	4.4–182	50.50	29.52–76.33	28.71	6.07–48.79
Mn	1.36	1.04	0.15–4.47	1.24	7.2	1.1	0.16–82	-	-	0.78	0.16–4.94
Na	3.53	3.0	1.79–12.6	2.47	33	31	5.9–150	47.57	34.61–63.94	15.30	2.46–92.73
Ni	0.14	0.13	0.02–0.22	0.05	-	-	-	-	-	0.1296	0.0503–0.3875
P	39.2	33.4	17.7–79.9	17.4	-	-	-	-	-	-	-
Pb	0.54	0.42	0.05–2.10	0.5	-	-	-	0.05	ND-0.21	0.0064	0.002–0.0176
Sr	0.10	0.10	0.04–0.28	0.06	-	-	-	0.03	ND-0.12	-	-
Zn	0.80	0.73	0.18–1.90	0.53	3.3	2.3	0.31–15		49–2495	3.43	0.62–19.17

According to the Food and Agricultural Organisation and World Health Organisation (FAO/WHO) codex Alimentarius CXS 193–1995 [52], the maximum concentrations of As, Cd and Pb permitted in food, as recommended by the Codex Alimentarius Commission, are different for different foods (there are no national standards for potentially toxic elements in food, or honey and pollen particularly, in Kosovo). For As, the permitted maximum concentration in foods ranges from 0.1–0.5 mg/kg; for Cd, the permitted concentration range is 0.003–2 mg/kg; and for Pb, it is 0.03–0.4 mg/kg. Except for the Pb median concentration (0.42 mg/kg), which is only a little higher than the maximum permitted by the FAO/WHO recommendation (0.4 mg/kg), As and Cd medians were quite lower than the maximum concentrations recommended by FAO/WHO for these metals. However, the Pb concentrations in many sampling sites exceeded the maximum recommended value of 0.4 mg/kg, as in the following: location No. 1 (0.47), 6 (1.14), 7 (0.65), 8 (0.7), 10 (0.78), 11 (2.1), 12 (0.55) and 16 (0.46). 

**Figure 3:**
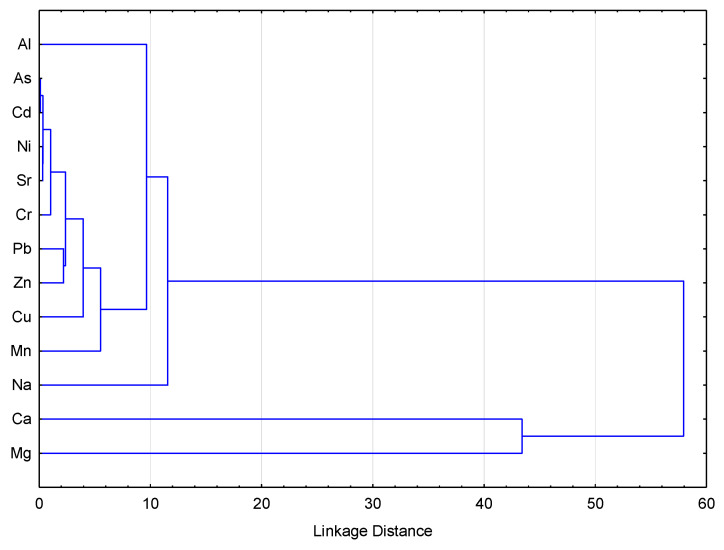
Hierarchical cluster dendrogram for elements in honey.

A hierarchical clustering analysis is presented in Figure 3, in which four clusters can be identified. Potassium stands out among all elements and forms the first cluster (not presented in dendrogram). It was found to be the most abundant element in honey with a median of concentration 775 mg/kg, followed by P (33.42 mg/kg); the origin of these elements is natural, even though fertilizers may have contributed to such high concentrations. Ca (15.7 mg/kg) and Mg (14.4 mg/kg) fall in the cluster two. The third cluster is made up of Na, Mn, Cu, and Al, while the fourth contains Zn, Pb, Cr, Sr, Ni, Cd, and As. Cluster three is probably of geogenic origin; these elements are absorbed by the root systems of plants over which the bees have been foraging, although some atmospheric provenance is also possible in the form of windblown dust. The fourth cluster is mostly anthropogenic. The highest concentrations of Cu, Zn, Pb, Cr, Ni, were mostly observed in samples around the cities of Mitrovica and Zveçan, where lead and zinc smelting sites are located, indicating obvious anthropogenic origin. Moreover, Al, As, Cd, (although not all in the same cluster) tended to be more concentrated in honey samples 7 to 13, which were closer to industrial ore processing facilities around the city of Mitrovica.

### 3.3. Pollen

The basic statistics for elements are presented in Table 7. The maximum concentrations of potentially toxic elements in honey bee pollen were in the following order: Zn (31.7) > Cu (7.65) > Ni (3.16) > Pb (1.69) > Cr (0.28) > Co (0.23) > Cd (0.15) > As (0.02), and the medians: Zn (26.5) > Cu (6.63) > Ni (2.0) > Cr (0.28) > Pb (0.2)> Co (0.12) > Cd (0.02) > As (0.01). Zinc was found to be the most abundant from among the selected potentially toxic elements in pollen, and was quite evenly distributed. Except for sample 3, in which the concentration of Zn was 16.6 mg/kg, all other samples had concentration ranges of 23.7–31.7 mg/kg. The distribution of copper was also quite homogeneous, with concentrations laying in the range of 5.66–7.65 mg/kg. The highest concentration of Pb was found in samples 4 (1.69 mg/kg) and 5 (0.91 mg/kg), whereas other samples contained 0.25 mg/kg or less. Cr was only detected in sample 3; in other samples, its concentration was too low to be quantified. 

Minerals are permanent constituents of pollen, and elements such as K, Ca, P, Mg, Zn, Fe, Mn, Cu, Na and Cr, are frequently reported [53,54,55]. Some research in Serbia [53] and Turkey [54] revealed that the most abundant elements in pollen are K, P, Ca, and Mg; see Table 7. In this study, the minimum and maximum concentrations of the same elements in mg/kg were 2902–4484 (K), 2503–4517 (P), 1018–1753 (Ca), and 393–762 (Mg). The mean concentrations in mg/kg of Zn (26.01), Cu (6.6), and Cr (0.28) were lower than those found in Turkey, i.e., Zn (29.15), Cu (10.4), and Cr (0.79), whereas mean Ni (2.11) in the Mitrovica region was higher than that in Turkey (0.51) and Serbia (0.76). The mean concentrations found in Mitrovica for Pb were higher than those in Turkey, whereas Zn had a higher mean than in Turkey. The maximum concentrations of Pb, Zn, and Cu in Jordan exceeded those in Mitrovica, whereas the maximum concentration of Ni was higher in the Mitrovica region. According to FAO/WHO codex Alimentarius CXS 193–1995 [52], the maximum concentration of As measured in the region of Mitrovica was under the minimum concentration permitted in some foods, i.e., 0.01 mg/kg. Cd and Pb exceeded the minimum concentrations set for some foods, i.e., 0.03 mg/kg for Pb and 0.003 mg/kg for Cd, but were lower than their permitted maximum of 2 mg/kg and 0.4 mg/kg respectively.

**Table 7:** Basic statistics of element concentrations in pollen (in mg/kg) in different countries.

Kosovo, 2019 (Present Work) *n* = 9					Turkey, 2017 [54] *n* = 24		Serbia, 2011 [53] *n* = 25	Jordan, 2017 [55] *n* =22
	Mean	Median	Range	STD	Mean	Range	Mean	Range
Al	33.2	31.7	8.96–57.7	16.2	-	-	38.6	-
As	0.01	0.01	ND-0.02	0.008	0.391	0.006–1.035	-	<0.02
Ba	4.66	4.72	3.47–6.25	1.12	-	-	1.22	-
Ca	1234	1194	1018–1753	215	862.435	491.85–1472.10	1425	-
Cd	0.05	0.02	0.02–0.15	0.05	0.069	0.006–1.035	0.067	<0.005
Co	0.13	0.12	0.06–0.23	0.05	-	-	0.047	-
Cr	0.28	0.28	0.28	-	0.793	0.124–1.595	0.26	-
Cu	6.61	6.63	5.66–7.65	0.59	10.418	3.728–14.994	7.8	0.032–11.388
Fe	59.0	59.4	22.0–94.3	22.5	203.165	30.719–725.36	70.1	-
K	3891	4026	2902–4484	428	1945.87	992.107–2894.15	3391	-
Mg	577	578	393–762	120	669.7	271.1–1278.3	749	641.38–1575.18
Mn	13.2	12.8	8.83–18.7	3.10	29.33	8.15–201.04	21.33	-
Na	37.0	37.6	21.3–45.3	8.59	-	-	21.6	-
Ni	2.11	2.00	0.98–3.16	0.82	0.51	0.02–1.76	0.76	<0.01–2.839
P	3633	3780	2503–4517	607	2659.7	795.9–5247	-	-
Pb	0.43	0.20	0.14–1.69	0.53	0.193	ND-0.479	-	0.03–2.567
Sr	3.09	3.27	1.99–4.50	0.97	-	-	1.38	-
Zn	26.0	26.5	16.6–31.7	4.53	29.15	14.83–39.08	23.7	25.24–77.022

**Figure 4:**
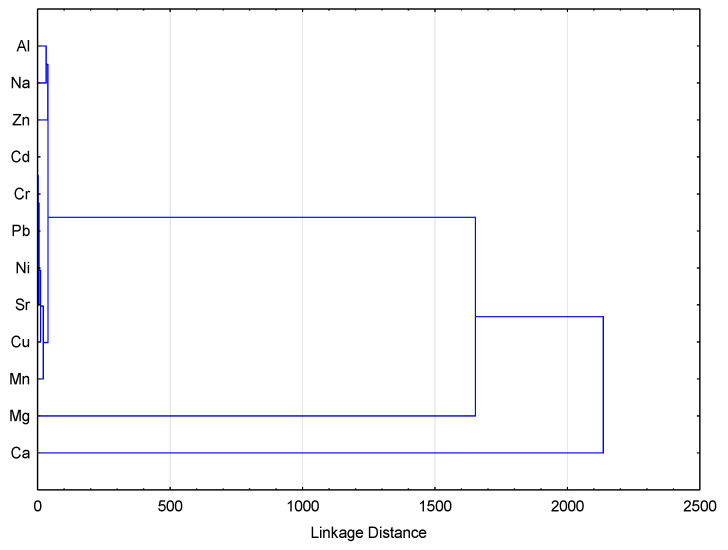
Hierarchical cluster dendrogram for elements in pollen.

A cluster analysis with Ward’s method revealed three distinct groups of elements in pollen (Figure 4). The first group was made up of K and P (not shown in the cluster), the second comprised Ca and Mg, and the largest group was constituted of Mn, Cu, Sr, Ni, Pb, Cr, Cd, As, Zn, Na, Al. The first and second groups were of natural origin. Group three included elements which are not used during in industrial process in the area of investigation. However, Pb and particularly Zn are indicators of pollution because of mining and ore processing activities, as are elements which are associated with their ores. An important pathway for the pollution of pollen with anthropogenic elements can be atmospheric deposition, since pollen is exposed to open air and elements can be transported as fine dust onto flowers [32].

### 3.4. Correlations between Three Types of Samples

**Figure 5:**
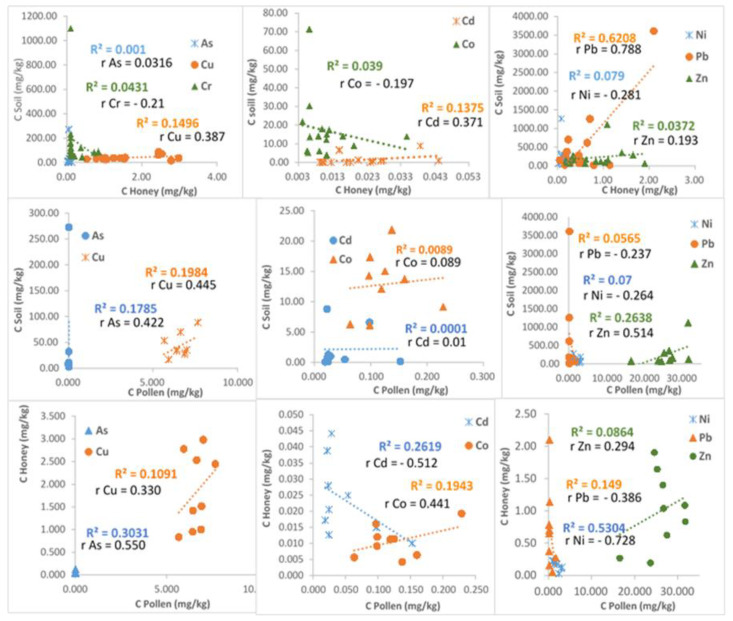
Pearson’s correlation coefficients of selected element concentrations among three sample types: soil, honey, and pollen.

According to the results shown in Figure 5, as expected, the highest values of all the potentially toxic elements were found in soil (vertical axes in the first two rows of the plots). Also, a higher concentration of a given metal in soil is not necessarily reflected in pollen or honey. For example, although Cu concentrations in soil were lower than those of Ni and Pb, in honey, they were higher. Also, if one element is found at higher concentrations in soil, it may be present at lower concentrations in honey. In Figure 5, the Pearson’s coefficients show that there was no correlation for As for soil–honey, Co for soil–pollen, and Cd for soil–pollen. There were 11 weak correlations, six moderate, and only two strong correlations. The strongest correlation was that for Pb between soil and honey, with a value of 0.788, and for Ni in the pair honey–pollen, with a value of 0.728. Soil–pollen and honey–pollen were the pairs with the greatest number of significant correlations. Eight correlations were negative, despite the fact that in all cases, no correlation or positive correlation was to be expected. The weak and negative correlations between concentrations of heavy metals in soil, honey and pollen can be attributed to the complexity of honey and pollen collection by bees. The distribution of heavy metals in soil is quite heterogeneous, and there is a large variety of plants which can absorb different elements in different quantities through their root systems [56]. Honey, and particularly pollen, are effected very much by dust. Bees forage in places such as dumps, when they can take water or other materials which may be heavily polluted with heavy metals, leading to the contamination of honey and pollen which is otherwise situated in a not heavily polluted area. 

## 4. Conclusions

An investigation into the concentrations of certain elements in soil, honey, and pollen was conducted in the region of Mitrovica, Kosovo. Extremely high concentrations of Pb, Zn, and Ni were found around the lead and zinc smelting sites in the towns of Mitrovica and Zvecan. As and Cr were also present in very high concentrations at locations close to an ore processing facility in Mitrovica. Pollution indices showed values indicating not polluted areas, moderately polluted areas, and extremely polluted ones, particularly with lead and zinc.

Cluster analyses revealed groups of elements related to sources of pollution, and also those that are usually expected to be of geogenic origin. It can be said that the soil in the region of Mitrovica, mostly around the industrial facilities, is highly polluted with potentially toxic elements. In honey and pollen, lead concentrations were concerning, and zinc was particularly abundant in pollen. The correlations between types of samples for each selected potentially toxic element were mostly weak and moderate. There were, however, important correlations indicating some pollution transfer from one environmental compartment to another. Also, since this is the first research on honey and pollen in the entire region of Mitrovica, more research should be conducted in order to more thoroughly evaluate the contamination situation of these two natural products. The lead/zinc smelting sites in Mitrovica have not been in operation since 2000; however, the ore concentration process should be improved to reduce dust emissions. Clearly, a strong source of pollution are tailings dumps, as fine particles are transported from them toward the surrounding environment. To prevent the spread of pollution, such should be covered with unpolluted soil or concrete. Another possibility is to transport the tailings into the cavities created in more remote areas during gravel excavation, and then to cover them with soil.

## Figures and Tables

**Table 3:** Contamination factors (*CF*) of selected elements for each sampling site.

No	As	Cd	Co	Cr	Cu	Ni	Pb	Zn
1	0.29	0.67	0.54	0.92	2.75	6.42	3.03	1.38
2	0.92	0.92	2.31	2.55	4.77	16.9	2.93	2.35
3	1.60	43.9	1.76	0.89	4.11	3.99	1.09	1.40
4	0.38	3.13	1.17	1.48	2.35	3.89	3.06	1.65
5	1.24	1.17	2.80	2.72	2.57	10.6	6.49	2.35
6	1.17	0.67	0.78	0.76	1.27	1.66	0.88	1.34
7	1.00	8.53	1.55	1.30	2.06	3.79	26.7	5.67
8	38.9	58.7	1.93	1.74	5.40	6.62	54.7	6.88
9	0.73	0.64	0.80	1.38	2.77	4.68	7.94	3.26
10	1.16	6.20	2.23	3.39	2.74	15.4	0.67	1.89
11	4.57	6.60	1.83	1.99	6.83	5.58	157	21.5
12	0.83	0.99	0.68	1.58	2.45	3.36	4.37	5.33
13	1.52	5.33	1.79	1.66	2.19	4.90	16.4	2.83
14	0.76	5.11	9.18	18.32	2.98	70.2	30.5	5.21
15	1.53	1.51	3.91	3.84	3.15	14.3	11.0	3.04
16	1.55	2.37	1.81	2.06	2.09	4.95	12.11	3.04

**Table 4:** Degree of contamination (*Cd*), modified degree of contamination (*mCd*), and pollution load index (*PLI*) for each sampling site.

No	*Cd*	*mCd*	*PLI*
1	16.0	2.00	1.28
2	33.6	4.21	2.69
3	58.8	7.35	2.69
4	17.1	2.14	1.77
5	29.9	3.74	2.87
6	8.54	1.07	1.02
7	50.6	6.33	3.46
8	175	21.9	10.0
9	22.2	2.78	1.91
10	33.7	4.21	2.71
11	206	25.7	7.83
12	19.6	2.45	1.90
13	36.7	4.58	3.24
14	142	17.8	8.26
15	42.3	5.28	3.89
16	30.0	3.75	2.91

**Table 5:** Geoaccumulation index (*Igeo*) of selected elements for each sampling site.

No	*I*_geo_(As)	*I*_geo_(Cd)	*I*_geo_(Co)	*I*_geo_(Cr)	*I*_geo_(Cu)	*I*_geo_(Ni)	*I*_geo_(Pb)	*I*_geo_(Zn)
1	−2.388	−1.170	−1.47	−0.70	0.88	2.10	1.02	−0.12
2	−0.711	−0.705	0.62	0.76	1.67	3.49	0.97	0.65
3	0.091	4.872	0.23	−0.75	1.45	1.41	−0.46	−0.10
4	−1.991	1.063	−0.36	−0.02	0.65	1.37	1.03	0.14
5	−0.274	−0.363	0.90	0.86	0.78	2.81	2.11	0.65
6	−0.359	−1.170	−0.93	−0.98	−0.24	0.15	−0.77	−0.16
7	−0.589	2.508	0.05	−0.20	0.46	1.34	4.16	1.92
8	4.697	5.290	0.36	0.21	1.85	2.14	5.19	2.20
9	−1.032	−1.229	−0.90	−0.12	0.88	1.64	2.40	1.12
10	−0.367	2.047	0.57	1.18	0.87	3.36	−1.16	0.34
11	1.608	2.138	0.29	0.41	2.19	1.89	6.71	3.84
12	−0.858	−0.595	−1.13	0.08	0.71	1.16	1.54	1.83
13	0.017	1.830	0.26	0.15	0.55	1.71	3.45	0.92
14	−0.979	1.769	2.61	3.61	0.99	5.55	4.34	1.80
15	0.028	0.013	1.38	1.36	1.07	3.25	2.87	1.02
16	0.045	0.658	0.27	0.46	0.48	1.72	3.01	1.02
